# Prediction of drug-drug interaction events using graph neural networks based feature extraction

**DOI:** 10.1038/s41598-022-19999-4

**Published:** 2022-09-16

**Authors:** Mohammad Hussain Al-Rabeah, Amir Lakizadeh

**Affiliations:** Computer Engineering Department, University of Qom, Qom, Iran

**Keywords:** Machine learning, Biomedical engineering

## Abstract

The prevalence of multi_drug therapies has been increasing in recent years, particularly among the elderly who are suffering from several diseases. However, unexpected Drug_Drug interaction (DDI) can cause adverse reactions or critical toxicity, which puts patients in danger. As the need for multi_drug treatment increases, it's becoming increasingly necessary to discover DDIs. Nevertheless, DDIs detection in an extensive number of drug pairs, both in-vitro and in-vivo, is costly and laborious. Therefore, DDI identification is one of the most concerns in drug-related researches. In this paper, we propose GNN-DDI, a deep learning-based method for predicting DDI-associated events in two stages. In the first stage, we collect the drugs information from different sources and then integrate them through the formation of an attributed heterogeneous network and generate a drug embedding vector based on different drug interaction types and drug attributes. In the second stage, we aggregate the representation vectors then predictions of the DDIs and their events are performed through a deep multi-model framework. Various evaluation results show that the proposed method can outperform state-of-the methods in the prediction of drug-drug interaction-associated events. The experimental results indicate that producing the drug's representations based on different drug interaction types and attributes is efficient and effective and can better show the intrinsic characteristics of a drug.

## Introduction

Recently, it became so popular to cure difficult diseases such as cancer using drug mixes or so-called Polypharmacy. It is a good approach, especially among the elderly who suffer from several diseases, using the synergistic effects of drug interactions. However, unplanned DDIs could risk a patient's life because they may cause side effects or perhaps dangerous toxicity. As the need for multidrug therapy increases, the detection of DDI becomes much more necessary^1,2^. However, the diagnosis of DDI on a large number of drug pairs, both in vitro and in vivo, is costly and time-consuming^3^. Therefore, detecting DDIs is one of the main concerns in pharmaceutical research^4^. Detecting possible DDIs decreases the incidence of unexpected drug interactions and reduces drug production costs. It also can optimize the drug creation process. Therefore, the research of DDIs and adverse drug reactions (ADRs) is necessary for drug production and clinical applications, specifically for concomitant drugs^5^.

The explosive growth of large-scale and high-precision biological data has led to the formation of a research field called computational pharmacology. This data creates the opportunity for systematic analysis of various data. Analyzing this data can be useful to improve drug development and reduce the risk. The interactions are very popular in biological processes as bonds within a chemical compound. Therefore, networks are usually used to represent biological data. The emergence of this biological network requires new computational tools for analysis^6^. Thus, new studies have tried to address this shortcoming.

Recently, a large number of researchers in the graphical data structure field has led to a high level of promotion of graphical data structure analysis techniques. Indeed, there is a lot of attention on deep learning and its applications in this field. However, many researchers have presented a method for computing the weighted average for node neighbor information based on neural network processing methods. These graphic data structure processing models, using neural networks, are known as Graph Neural Networks (GNNs)^7^. This method extended the current neural network for processing graphical data structures.

In general, there are four popular approaches in the DDI prediction field: Similarity-based methods, Matrix Factorization-based methods, network analysis-based methods and Deep Learning-based methods. Similarity-based methods are based on the similarities between drugs and proteins, or drugs and diseases, and vice versa. They employ a classical classification model, such as SVMs, regular least squares, logistic regression, and random-forest to complete the prediction task^8^. Gottlieb et al.^9^ calculated feature vectors based on seven types of drug-drug similarities to represent drug-drug pairs and then used a weighted logistic regression model to predict DDI. Cheng et al.^10^ combined a variety of drug-drug similarities to represent drug-drug pairs and utilized five classifiers to construct the prediction models. Dang et al.^11^ adopt a machine learning model to predict DDI types for histamine antagonist drugs using two similarity matrices as inputs. Then employ various classification algorithms such as Naive Bayes, Decision Tree, Random Forest, Logistic Regression, and XGBoost for DDIs prediction. Song et al.^12^developed a machine learning model using support vector machines (SVMs) based on several similarity matrices and then employed them as the input vector of the SVM.

Matrix Factorization is extensively used for data analysis. It factorizes the data matrix into a matrix with a smaller dimension. Then rebuild the adjacency matrix to determine DDIs. Yet, it maintains the complex structure and latent topological properties. Common Matrix Factorization has many forms, like (SVD) and graph factorization. Zhang et al.^13^ propose a matrix factorization method called (MRMF) which uses known DDIs and drug feature-based manifold to predict possible drug-drug interactions. Shi et al.^14^ develop a matrix factorization method named (BRSNMF) to divide drugs into communities and identify enhancive and degressive DDIs in the cold-start scenario. Rohani et al.^15^ collects several drug similarities and then utilized Integrated Similarity-constrained matrix factorization to identify DDIs. However, modern studies concentrate on developing different high-order data proximity matrices to maintain graph structure. For example, GraRep^16^ adopts the network high-order proximity and makes factorization by building k-step transition probability matrices.

Network-based methods employ network structure to construct relationships among biological and biomedical entities for predicting potential interactions^8^. Random walk-based methods are a famous approach in this field. These methods employ random walks in the networks to construct a node sequence. Then the method learns node embeddings using the word2vec model. One of the earliest models is DeepWalk^17^, which executes trimmed random walks on a network. Next,^18^ struc2vec is proposed for more acceptable modeling of the network structure. Especially, struct2vec can describe multi-layer weighted graph constructs. Lee et al.^19^ build a heterogeneous biological network using a combination of several databases and interaction data. Then adopted a method to calculate the relation strength between two drugs and discover paths of drug-drug pairs. Huang et al.^20^ suggested a metric that calculates the relations strength of the network called 'S-score' to find possible PD DDIs. Lee et al.^21^ produce a global graph by employing a random walk with a restart algorithm and using the global information for prediction.

Deep learning is a recently popular branch of artificial neural networks that learns a sequential representation layer of features during the learning process. This approach is used in many fields effectively such as computer vision, NLP, and bioinformatics^8^. Many Types of neural networks were established in the graph embeddings area, such as autoencoder^22^, MLP^23^, and GAN^24^. Embedding network-based data is modeling a set of entities (nodes) and their links (edges). DeepWalk is the first model of processing graphical data using a deep learning approach. Many algorithms proposed motived by DeepWalk like node2vec and Metapath2vec. Also, there is recent progress in deep learning-based drug repositioning such as HINGRL^25^, MGRL^26^ and^27^. Lately, a lot of studies in the field of graphical data structure have been extremely advanced for processing network data structure^28^. Several researchers have developed a neural network method for computing a weighted average of information for each node's neighbor. These methods that employ neural networks for computing graphical data structure are generally known as Graph Neural Networks (GNNs). The GNN concept was initially introduced in 2009^7^ which expanded the current neural network for computing graphical structure data. Several GNN methods for graphical data structure were proposed, containing Graph Auto-encoders (GAEs)^29,30^, Graph Convolutional Neural Networks (GCNs)^17,18^, and Graph Recurrent Neural Networks (Graph RNNs)^23,31^. Moreover, deep learning-based methods have been commonly used in the biomedical area^32,33^ and have earned very good results. Karim et al.^5^ built a knowledge graph from several databases and used knowledge graph embedding to generate a drugs feature vector. Then employs a CNN-LSTM model for DDI prediction. Feng et al.^3^ proposed a technique called DPDDI to predict DDIs by collecting the drug's features from the DDI network with GCNs. Then uses the deep neural network model for prediction. Liu et al.^34^ present a framework named DDI-MDAE supported by multi-modal deep auto-encoders using shared latent representation to identify DDIs. Lin et al.^35^ present an end-to-end framework, called KGNN. This framework can effectively extract the drugs and their potential neighbors.

Normally, current methods are developed to predict whether drugs interact or not. However, DDIs may show different biological effects or events. Predicting DDI-associated events is an important and difficult task, and has acquired some attention^36^. Ryu et al.^37^ presented a deep learning approach based on drug chemical substructures to predict 86 crucial DDI types. Feng et al.^38^ proposed a novel end-to-end deep learning-based predictive method called MTDDI to predict DDIs as well as their types. Deng et al.^4^ presented a multimodal deep learning framework that employed multiple drug features to predict 65 categories of DDI events. Even though the above methods have created strong efforts in event prediction but there is a space for advancement.

The DDI network can provide vital information about drugs interactions. Furthermore, using an attributed heterogeneous DDIs network that presents the drug's interaction types along with the drug features can better demonstrate the intrinsic characteristics of a drug. However, it is challenging to integrate various features effectively because the drug features might be correlated and contain redundant information. Directly combining various feature vectors is a common strategy, but we need a more effective framework for aggregating the features.

Here, we proposed a method for predicting DDI and their type (event) based on attributed heterogeneous graph embedding and a deep learning approach. The method consists of two stages. In the first stage, the data is collected and used to make four feature matrices (Chemical structure, Target, Enzyme, and Pathway) and one drug-drug matrix. Then the drug-drug matrix is used to build a heterogeneous network of drugs as nodes. The feature matrices after preprocessing are used in the network as node attributes. In this approach, we use the attributed heterogeneous network representation technique to integrate different drug properties in each type of drugs interactions and creates drug embedding vectors. The second step begins with the preparation of the embedding vector for each drug obtained from the previous step. Using one of the concatenation methods the feature victor of the drug pairs is obtained. Finally, a fully connected neural network uses these embedding vectors as input to predict the drug interaction types.

The proposed method is summarized in the following steps:

Step 1: Integrating data sources and extracting embedding vectors (final feature vectors):

- Gathering drug data and calculating similarity matrices for each drug feature.
- Building an attributed heterogeneous graph as an Integration graph.
- Calculating drug embedding matrices by embedding an attributed heterogeneous graph using a new GNN model.

Step 2: Predicting Drug–Drug Interactions (DDI) types:

- Reducing the dimensions of the matrix obtained from the previous step in the embedding process by merging the drug embedding vectors for each interaction type.
- Creating matrices of drug pairs by Integrating the embedding vectors of each drug pairs.
- Finally, the above vectors are given as input to a deep learning network to predict the type of drugs interaction.

## Experiments and results

### Evaluation metrics

There are two main tasks in DDI prediction, first is identifying the interactions among the drugs. The second is to determine what kind of interaction is between drugs. In this article, we employ k-fold cross-validation to evaluate the DDI prediction task. We randomly split the known drug-drug interactions into *K* subsets of equal size. Here we use fivefold (5-CV). In each fold, we use one subset as the testing set and keep the rest for training.

Here, we utilize different evaluation metrics to measure the prediction model performances. Our task is the multi-class classification work. We use accuracy (ACC), Area Under the Precision-Recall-Curve (AUPR), area under the ROC curve (AUC), F1 score and Precision and Recall as the evaluation metrics. We use micro metrics for AUPR and AUC and macro metrics for the others. The micro-scale studies the classes individually, but the macro-scale interacts with the sum or the whole, so the calculation is general. The difference between macro and micro scales is that the macro scale weighs all classes equally, while the micro-scale weighs each sample equally. If the number of samples is equal for each class, the micro and macro scales will have the same score. Here in this multi-class problem, micro-Precision, micro-Recall and micro-F1 are equal to accuracy.

### Parameter setting

In this section, we discuss the effect of using different values for hyperparameters that influences the performance of the proposed model. The model consists of two stages. Therefore, we discuss embedding dimensions in the first stage and vector integration methods in the second stage.

#### Effect of embedding dimension size

**Figure 1:**
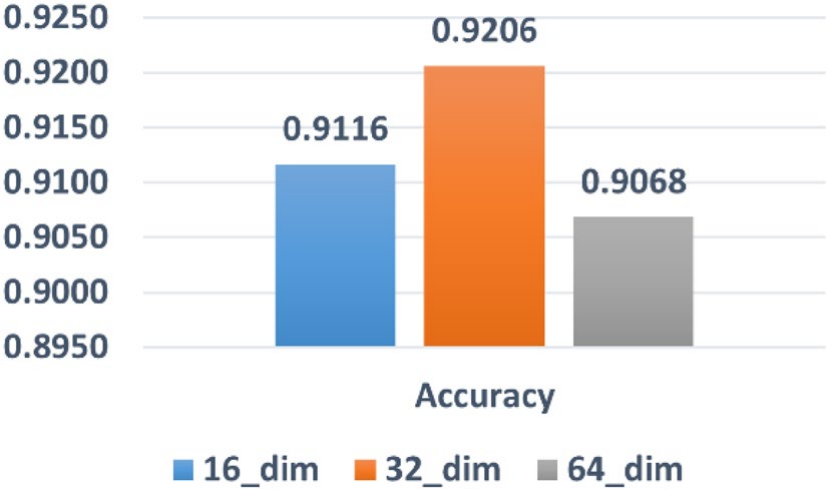
The effect of different values for embedding dimension in terms of model's accuracy.

Here, we evaluate the model performance using different sizes for embedding dimensions of the drugs. Figure 1 shows the performance of using different values for embedding dimensions. We found that the model with a vector size of 32 led to the best accuracy, which is probably due to the better representation of drugs. The embedding dimension with size 16 also shows good performance and is less time-consuming. Nevertheless, it achieves lower accuracy.

#### The effect of different integrating schema in terms of the model's accuracy

**Figure 2:**
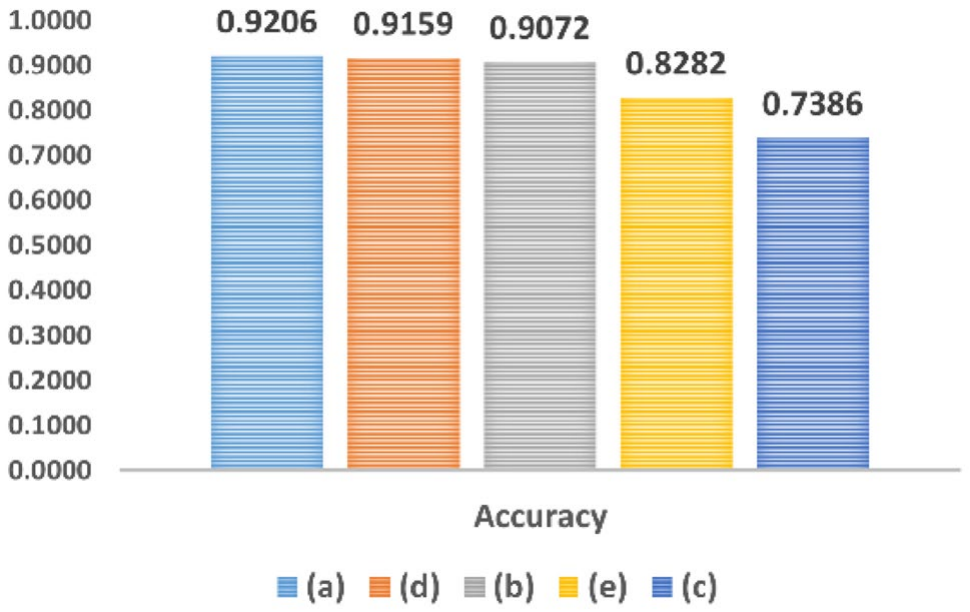
The effect of using different integrating schema in term of model's accuracy.

**Figure 3:**
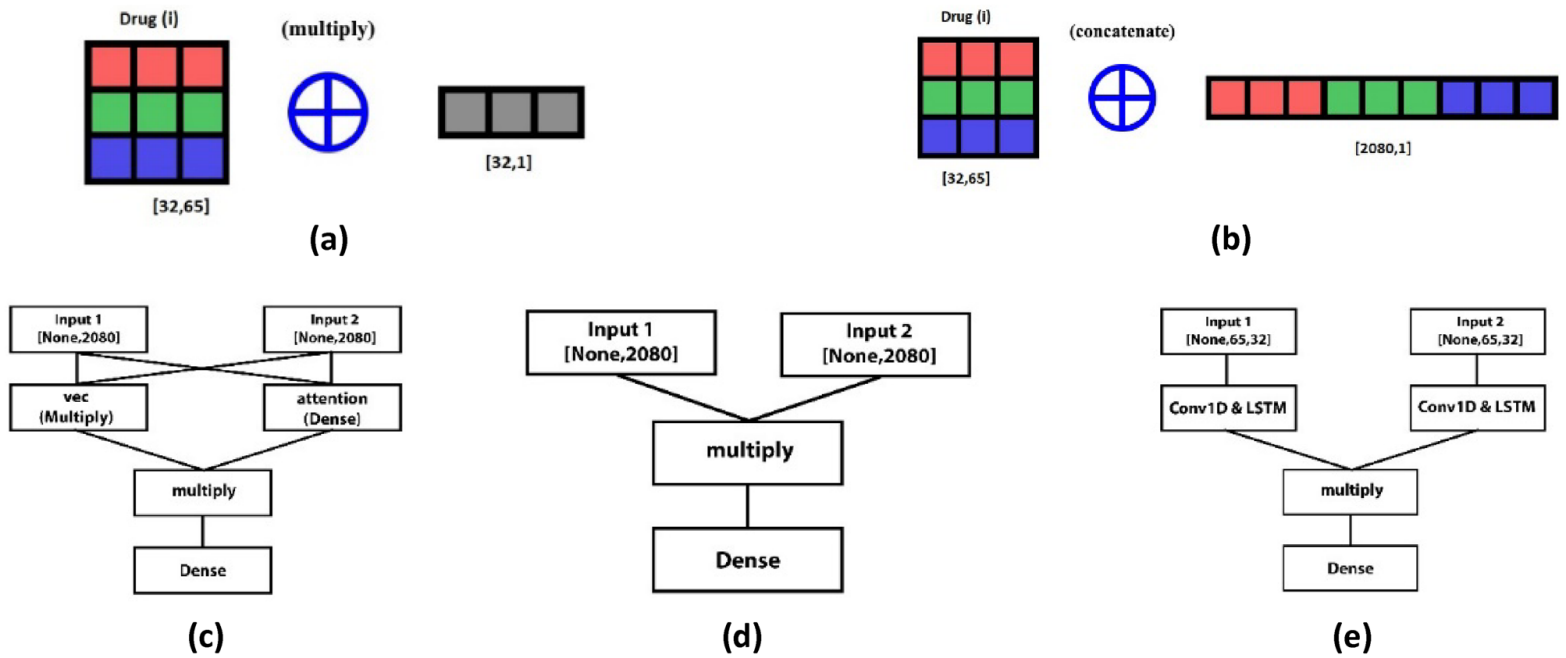
An overview of integration methods.

In this section, we discuss the effect of using different integration schemas of drug vectors. Integrating various features effectively is a difficult task because the drug features might be correlated and redundant. However, directly merging diverse feature vectors is a familiar strategy, but we need a more effective framework for aggregating the features. We test several aggregation schemas and choose the best one for the task. According to Table 1. the integration schema (a) shows better performance and achieves the best accuracy. This schema combines each drug embedding vector in all event types (interaction types) using (np.concatenate) as explained in the Eq. (1).

1 $$ F_{i} = \left[ {v_{i,1} , \ldots , v_{i,t} } \right] $$

2 $$ F_{i,j} = F_{i} \odot F_{j} $$

3 $$ F_{i,j} = \left[ {F_{i} ,F_{j} } \right] $$

where the embedding vector $v$ in certain edge type $t$ for certain drug $i$ is $v_{i,t}$ and the one-dimensional feature vector for drug $i$ is $F_{i}$. Then multiplies two vectors of drugs pair ($F_{i}$ and $F_{j}$ ) using (np.multiply) as shown in the Eq. (2). Where the feature vectors of the drugs pair are $F_{i,j}$. This schema led to the best accuracy. However, the integration schema (b) performs well but it consumes a lot of storage space and time and achieves less accuracy than (a). The integration method (b) uses the same way of combining each drug embedding vector in all event types using (np.concatenate). But then it uses (np.concatenate) again to merge the vectors of drugs pair as shown in the Eq. (3). The integration method (d) achieves the second-best accuracy after (a) but it is also time-consuming. Finally, Table 1. and Fig. 2 shows the effect of using different integrating approaches in term of the model's accuracy. Figure 3 shows an overview of integration methods.

**Table 1:** The effect of different integrating schema in term of model's accuracy.

Integration method	Integration Description	Accuracy
(a)	This method combines each drug embedding vector in all event types using (np.concatenate) as explained in the Eq. (1). Then multiplies two vectors of drugs pair using (np.multiply) as shown in the Eq. (2)	0.9206
(b)	This method combines each drug embedding vector in all event types using (np.concatenate). Then it merges the vectors of drugs pair using (np.concatenate) as shown in the Eq. (3)	0.9072
(c)	This method combines each drug embedding vector in all event types using (np.concatenate). Then the multiplication of the vectors of the drug pairs was performed using the multiplication method used in the article^39^	0.7386
(d)	This method combines each drug embedding vector in all event types using (np.concatenate). Then multiplies two vectors of drugs pair using (keras.layers.Multiply) during training as explained in the Eq. (2)	0.9159
(e)	Each two-dimensional embedding matrix of the drugs pair is given as an input to a neural network to make predictions. This neural network takes two two-dimensional matrices as input and produces one output. The model processes each matrix through several layers of conv1D and LSTM. Then the model uses the layer (keras.layers.Multiply) to multiply the output of the two LSTM layers. Then it passes the result through a series of (keras.layers. Dense) layers to make the prediction	0.8282

### The effect of using different drug features

We examined the proposed method in several cases based on using different drug features as input to make a more accurate evaluation. First, we implemented the proposed method on each feature matrix (similarity‌ matrices) separately. Then, we implemented the proposed method on a combination of feature matrices. Table 2 shows the results for using the feature matrices in different ways, as well as the results for all feature matrices combinations. We refer to the embedding of drugs interactions network with GD, and to the Enzyme, Target, Chemical structures and Pathways with E, T, S, and P respectively.

**Table 2:** Effect of using different data sources in terms of evaluation measures.

Dataset	${\text{Accuracy}}$	${\text{AUPR}}$	${\text{AUC}}$	${\text{F}}1{\text{ score}}$	${\text{Precision}}$	${\text{Recall}}$
$S$	0.8623	0.9136	0.9975	0.7324	0.7831	0.7006
$T$	0.8338	0.8979	0.9969	0.7084	0.7579	0.6788
$P$	0.8182	0.8876	0.9972	0.6875	0.7611	0.6495
$E$	0.6687	0.7384	0.9913	0.4105	0.4943	0.3714
$S + T$	0.8806	0.9192	0.9981	0.7625	0.8231	0.7283
$S + P$	0.8786	0.9188	0.9981	0.7611	0.8326	0.7223
$S + E$	0.8655	0.8939	0.9970	0.7263	0.8324	0.6821
$T + P$	0.8344	0.9004	0.9976	0.7012	0.7781	0.6660
$T + E$	0.8506	0.8860	0.9970	0.6974	0.7770	0.6564
$P + E$	0.8423	0.8809	0.9968	0.6664	0.7344	0.6279
$S + T + P$	0.8625	0.9202	0.9982	0.7330	0.7941	0.6950
$S + T + E$	0.8852	0.9208	0.9979	0.7585	0.8471	0.7182
$S + P + E$	0.8778	0.9153	0.9978	0.7321	0.8134	0.6905
$T + P + E$	0.8488	0.8956	0.9974	0.6967	0.7608	0.6591
$S + T + P + E$	0.8725	0.9178	0.9979	0.7361	0.8348	0.6938
$GD$	0.8894	0.9517	0.9987	0.7859	0.8803	0.7500
$GD + E$	0.8169	0.8912	0.9971	0.7668	0.8106	0.7544
$GD + P$	0.8443	0.9152	0.9978	0.8045	0.8811	0.7671
$GD + S$	0.8279	0.9017	0.9974	0.7053	0.8378	0.6453
$GD + T$	0.8605	0.9276	0.9980	0.7681	0.8589	0.7317
*GD* + *E* + *P*	0.8735	0.9403	0.9987	0.8116	0.8998	0.7672
*GD* + *E* + *S*	0.8692	0.9297	0.9983	0.8062	0.9084	0.7592
*GD* + *E* + *T*	0.8662	0.9340	0.9985	0.8071	0.8967	0.7754
*GD* + *P* + *S*	0.8833	0.9426	0.9987	0.7936	0.8994	0.7480
*GD* + *P* + *T*	0.8921	0.9498	0.9988	0.8341	0.9179	0.7963
*GD* + *S* + *T*	0.8863	0.9420	0.9986	0.7980	0.9146	0.7514
*GD* + *E* + *P* + *S*	0.9035	0.9584	0.9991	0.8359	**0.9432**	0.7833
*GD* + *E* + *P* + *T*	0.8982	0.9529	0.9990	0.8453	0.9049	0.8204
*GD* + *E* + *S* + *T*	0.9035	0.9582	0.9991	0.8428	0.9389	0.7958
*GD* + *P* + *S* + *T*	0.9067	0.9578	0.9991	0.8331	0.9307	0.7874
*GD* + *E* + *P* + *S* + *T*	**0.9206**	**0.9717**	**0.9993**	**0.8579**	0.9204	**0.8260**

Significant values are in bold.

**Figure 4:**
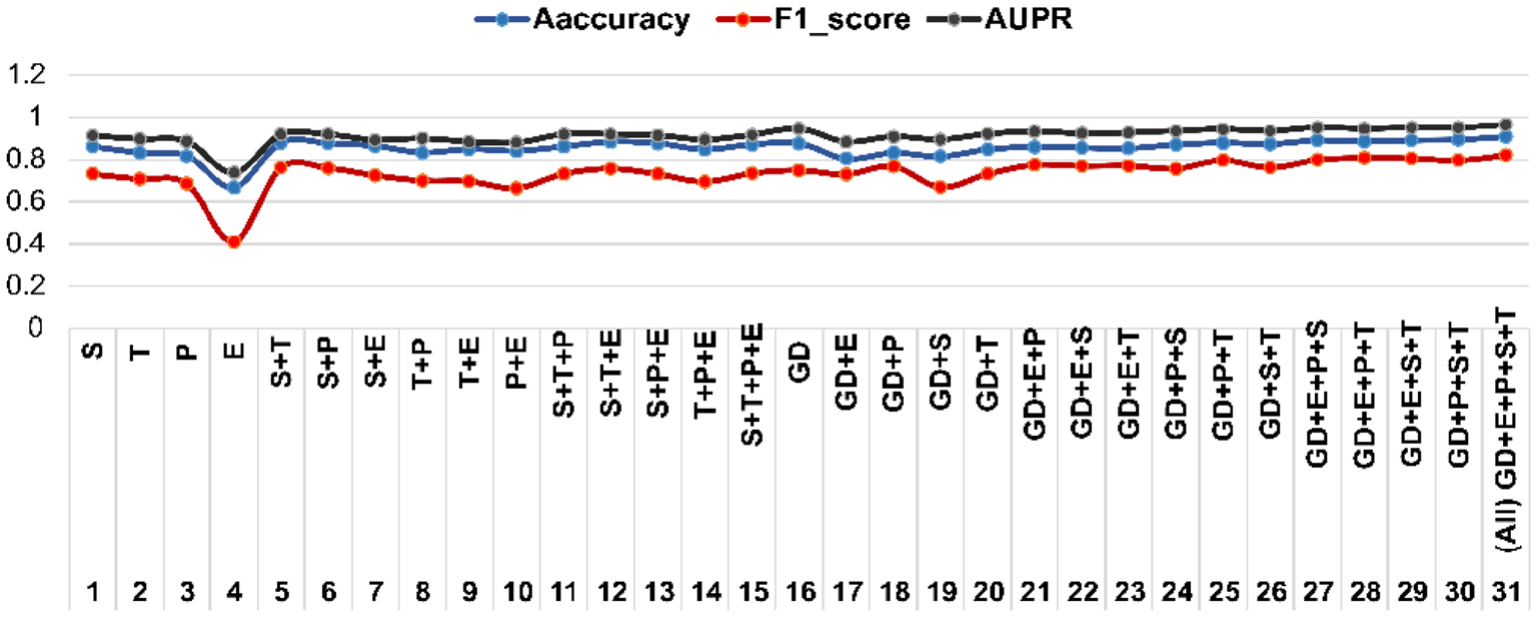
Effect of using different feature matrices in terms of evaluation measures.

The combination of different feature matrices has led to better results. The model performance using GNN models shows the efficiency in extracting and summarizing the drug's features from the network structure. Furthermore, using the embedding of drug interactions network alongside the features matrices show better performance for the model and the best result in AUC and AUPR values. The model using the enzyme matrix shows the lowest accuracy. While the Chemical Structure matrix has the highest accuracy in the individual evaluation of each matrix, which appears to be a more informative and good effect on explaining the interaction. In general, using drug network embedding alone shows significant improvement in accuracy. It is probably due to the presence of topological information in this network structure that led to better modeling of the drug's interaction. However, using the feature matrices as node attribute improves the model performance because of the information that these features add to the model. Figure 4 shows the integration of different feature matrices improved the accuracy. Also, using drugs network embedding along with the feature matrices as node attributes achieved the best result.

### Comparison with the other methods

We compared the proposed model with several state-of-the-art event prediction methods: DDIMDL, CNN-DDI, DANN-DDI and MDNN. We also consider many popular classification methods, i.e. random forest (RF), K-Nearest Neighbor (KNN) and Logistic Regression (LR). We compare the proposed model with these models to explain the advantages of utilizing the attributed heterogeneous network embedding method using drug features and the DDI edge list. Further, to show the impact of efficient aggregation schema. The DDIMDL model uses four similarity matrices of drug features as input. This model adopts four sub-network to learn cross-modality representations of drug-drug pairs. The DANN-DDI model after constructing multiple drug feature networks adopts an attention neural network to aggregate the learned drug representations and predict drug-drug interactions. We implement DANN-DDI according to the descriptions in^39^. CNN-DDI model first gathers the feature vectors from interaction matrices and calculates drug similarity. Then, it uses the features representation as input for the convolution neural network model to identify DDIs. We perform the CNN-DDI model based on the descriptions in^40^. The MDNN model develops a two-pathway framework. The framework includes a drug knowledge graph (DKG) based pathway and a heterogenous feature (HF) based pathway to produce drug multimodal representations. Next, the model employs a multimodal fusion neural layer to predict DDI events. We implement MDNN according to the descriptions in^41^.

**Figure 5:**
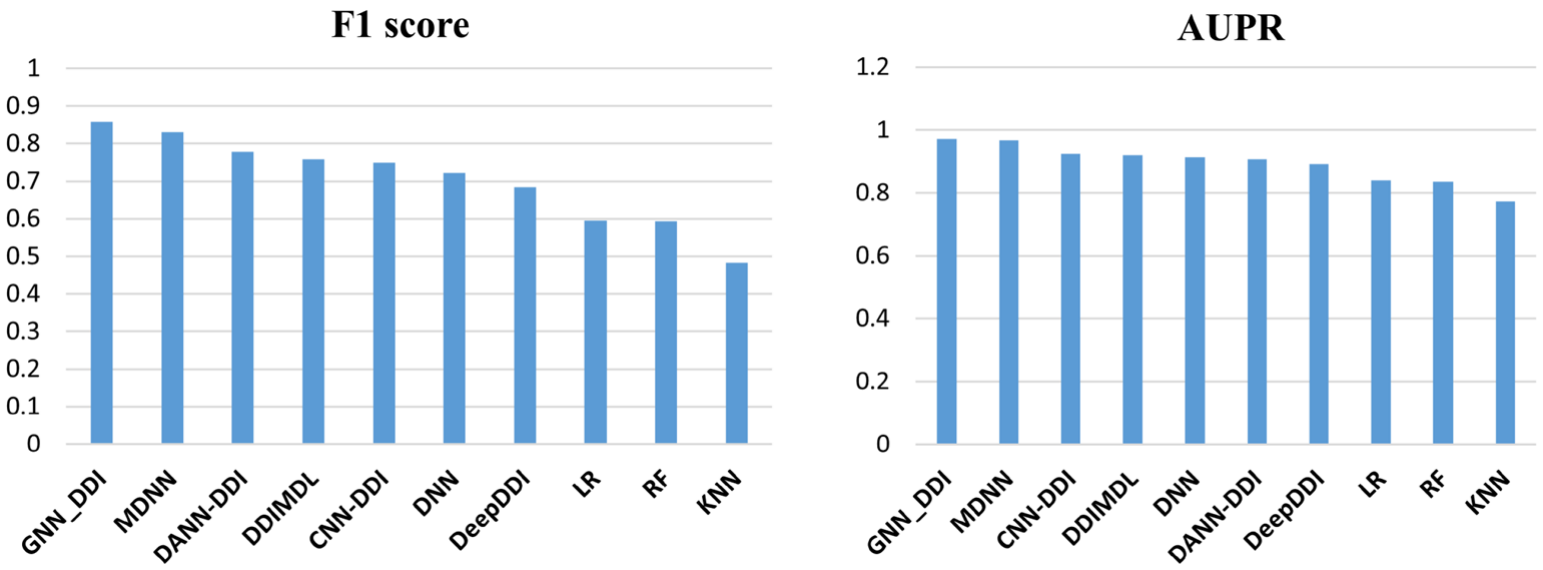
Comparison results of the proposed method with the other methods.

We use Table 3 to list all the prediction model's results. Figure 5 shows the performance of different models. The results show that the proposed model outperforms all of the comparison models in all metrics. The proposed model can overcome the imbalance challenge in the dataset and achieve the best AUPR score for the DDI event prediction task. Due to the imbalance in the dataset, the other models easily overfit. This shows the advantage of using the attributed heterogeneous network embedding method because the model extracts the drug representation in all different interaction types which can better describe the intrinsic characteristics of a drug. Furthermore, the proposed model tests several aggregation schemas and applies the best one for aggregating the features. Then, the model uses the joint sub-networks framework to combine the feature vectors of the drugs and predict the DDI events. The proposed model improved the prediction process based on AUC, AUPR and F1_score metrics and achieved 0.9992, 0.9717 and 0.8579, respectively. The model results during five folds show minimum accuracy of 0.9196 and average accuracy of 0.9211 and maximum accuracy of 0.9220. The results of the model in five folds are shown in Table 4.

**Table 3:** Results of comparison of the proposed method with the previous methods.

Method	${\text{Accuracy}}$	${\text{AUPR}}$	${\text{AUC}}$	${\text{F}}1{\text{ score}}$	${\text{Precision}}$	${\text{Recall}}$
**GNN_DDI**	**0.9206**	**0.9717**	**0.9992**	**0.8579**	**0.9204**	**0.8259**
MDNN	0.9175	0.9668	0.9984	0.8301	0.8622	0.8202
CNN‑DDI	0.8871	0.9251	0.998	0.7496	0.8556	0.722
DANN_DDI	0.8874	0.9088	0.9943	0.7781	0.8485	0.7421
DDIMDL	0.8852	0.9208	0.9976	0.7585	0.8471	0.7182
DeepDDI	0.8371	0.8899	0.9961	0.6848	0.7275	0.6611
DNN	0.8797	0.9134	0.9963	0.7223	0.8047	0.7027
RF	0.7775	0.8349	0.9956	0.5936	0.7893	0.5161
KNN	0.7214	0.7716	0.9813	0.4831	0.7174	0.4081
LR	0.792	0.84	0.996	0.5948	0.7437	0.5236

Significant values are in bold.

**Table 4:** The results of the proposed model in five folds (5 CV).

Metric	min	max	ave
Accuracy	0.9196	0.9220	0.9211
AUPR	0.9705	0.9717	0.9713
AUC	0.9991	0.9992	0.9992
F1_Score	0.8507	0.8606	0.8556
Precision	0.9113	0.9211	0.9180
Recall	0.8203	0.8349	0.8254

**Figure 6:**
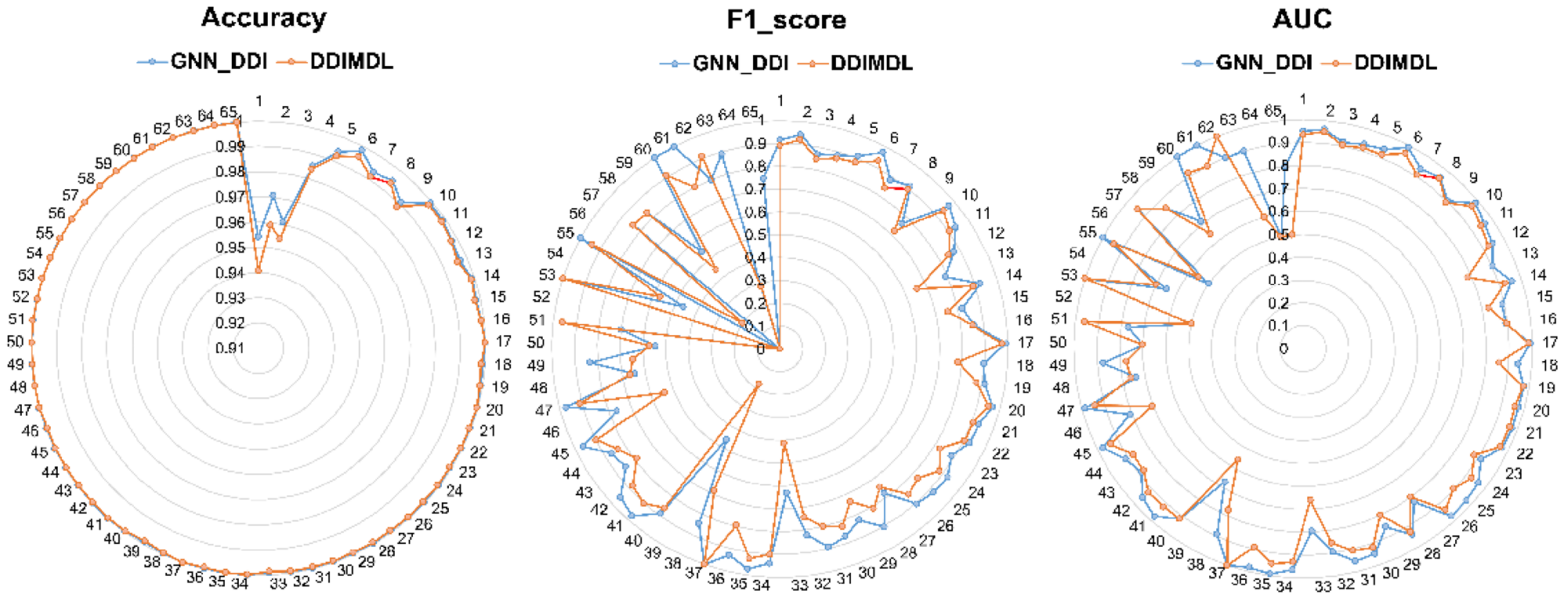
Results of the proposed method for detecting different events.

Figure 6 shows the efficiency of the proposed method in predicting different interactions type between drugs independently. Here we use the word event to address the interaction type between drugs. The model achieved good AUC and F1 scores in predicting most drug events. Except the event 39, which is wrongly classified as event 1. It may be because both events are related to drug metabolism. Also, the model for drug events from 51 to 65 achieved low metric scores in AUC and F1 scores, and it is due to the lack of samples. It has already been pointed out that the data is unbalanced, which is a big challenge and a regular problem in biological data. But the proposed method was able to deal with this imbalance in data.

## Conclusion

In this study, we construct a drug-drug heterogeneous network and several similarity matrices, such as drug-target, drug–chemical structure, etc. We use this network and the matrices in form of an attributed heterogeneous network to extract the drug feature vector using a GNN embedding method. The proposed model uses the drug network structure with the nodes attribute to generate drug embedding. Then, the proposed model integrates the drug feature vectors and finally adopts a fully connected sub-networks framework to predict the Drug-Drug Interaction type. We explain the dataset and evaluation metrics and discuss the results and evaluations of the proposed model. We apply five-fold cross-validation to the proposed model for evaluation. The model achieved 0.9220 as max accuracy and 0.9211 as average accuracy. The proposed model outperforms the existing DDI event prediction method. Also, we implement the model on each similarity matrix separately. Then we implement it on a combination of similarity matrices and report the results of predicting drug events. Further, we discuss the influence of using different hyperparameters in the model performance. We discuss utilizing various drug embedding dimensions and methods of integrating drug embedding vectors.

In conclusion, employing the attributed heterogeneous network embedding method can provide better drug representation in different drug interaction types and lead to better model performance. Also, using an effective aggregation schema and implementing a fully connected sub-networks framework can provide a powerful method to integrate various drug features. Furthermore, the experimental results indicate that this model outperforms the existing approaches. We can use the PU Learning strategy for future work to enhance the network positive samples by classifying the unlabeled data. Also, we can use a new approach to consider new drugs in the DDI event prediction process.

## Materials and methods

In this work, we propose a framework of two stages that combines several drug features to predict DDI-associated events, using attributed heterogeneous networks representation and aggregation schema with multiple deep neural networks. Firstly, it generates drug embedding from attributed heterogeneous networks using a GNN model. Next, it aggregates the feature vectors and uses multiple deep neural networks for DDI event prediction.

### Data collection

The data used in this research is derived from the study of Deng et al.^4^. Researchers in this study obtained and cleaned the required data from reputable databases such as DrugBank^42^ and KEGG^43^. This dataset includes four types of property or feature matrices for drugs: Chemical structure, Target, Enzyme, and Pathway. We obtained the pathway matrix from DrugBank and KEGG databases. But the rest of the matrices were collected from the DrugBank database. Each column in the features matrices represents the drugs. The rows represent specific drug properties (For example, the number of Enzyme types). The values of one and zero for each entity indicate the presence/absence of a specific property, respectively (for example, a certain enzyme for a particular drug).

**Figure 7:**
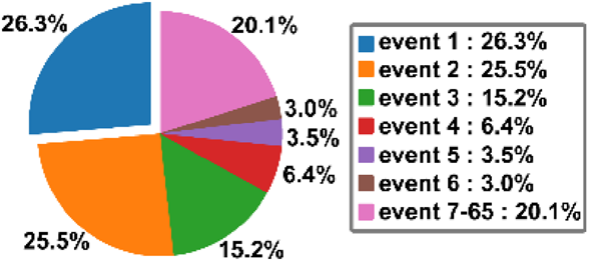
The difference in the number of samples between events.

The dataset provides a drug-drug edge list that includes 65 types of drug-drug relationships. The drug relationship refers to drug interactions. This database displayed drug interaction events as a quadruple structure: (drug A, drug B, mechanism, action). "Mechanism" means the effect of drugs in terms of metabolism, therapeutic effect, etc. "Action" indicates an increase or decrease in the effects. We employ the first two sections as an edge list of drug interactions and the second two sections as an interaction type or so-called event. The distribution of these events is not even, so the data is unbalanced. Figure 7 shows the distribution of the samples between events in the dataset. Therefore, the model under-fits simply in training, which is one of the main difficulties in this dataset. We use the edge list of DDI and one of the feature matrices to construct an Attributed Heterogeneous Graph. In this graph, the nodes refer to drugs and the links between them indicate 65 types of interactions. Table 5 shows a summary of these matrices.

**Table 5:** Types of properties in the dataset.

Type of data	
Drugs	572
Drug-Drug links	37,269
Target	1162
Enzyme	202
Chemical structure	881
Drug Pathway	957

### The first step of the proposed method

**Figure 8:**
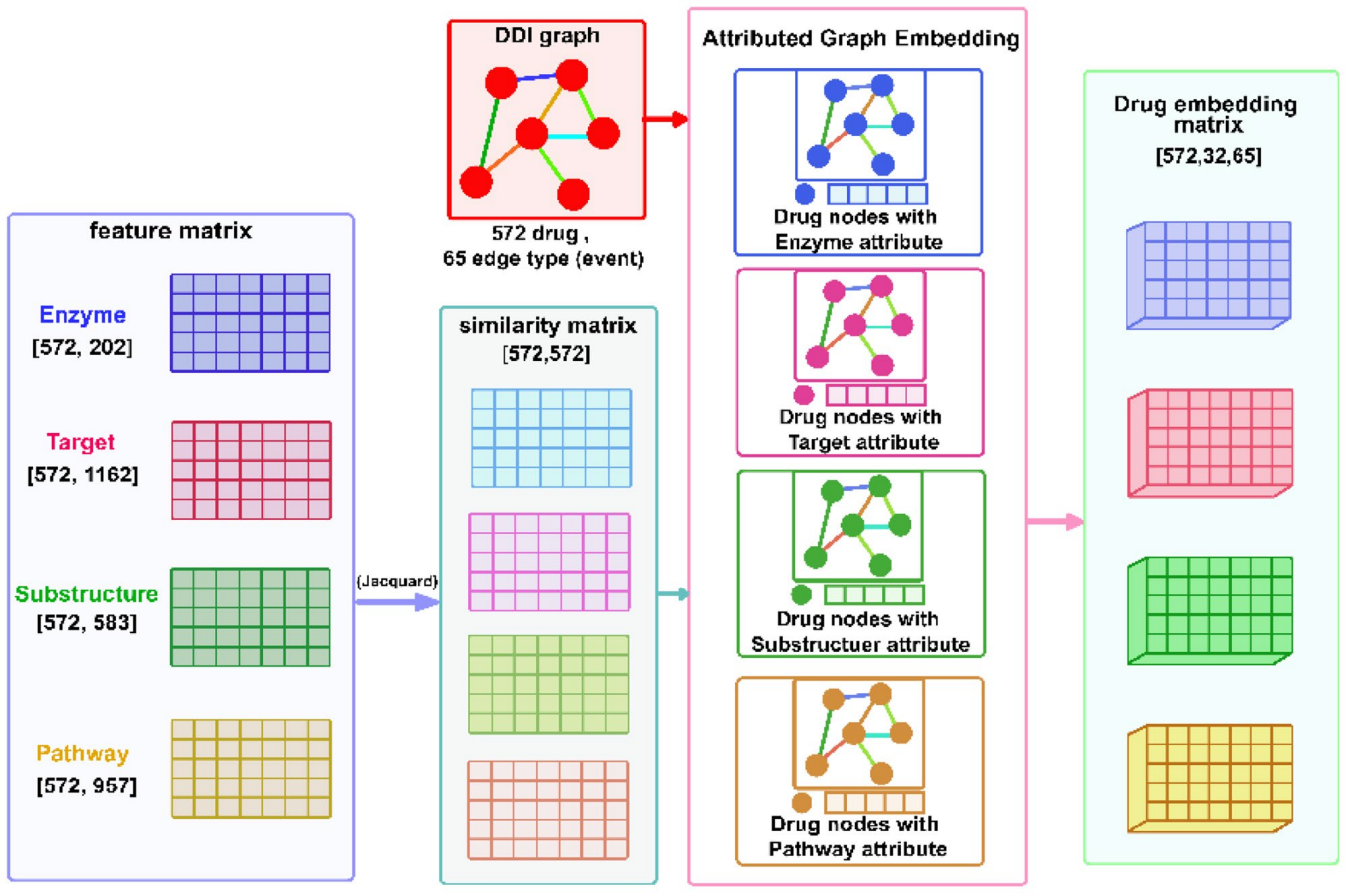
A view of the first step of the proposed method.

In the first stage of this approach, after preparing the attributed heterogeneous graph of drugs and feature matrices, we start the embedding process for each drug in each event type. At the end of this process, we will have an embedding matrix. In this matrix, each vector represents the embedding of that drug in a particular event type. Figure 8 shows a view of the first step of the method. Next, we discussed the details of each step in this phase.

#### Collect data and construct similarity matrices

Firstly, we collect five adjacency matrices from the information sources. A drug-drug matrix shows the interaction between two drugs and their event type. The feature matrices (drug-enzyme, drug-target, Drug–Chemical structure and drug-pathway) indicate the relationship between the drug and a particular feature. Then, after obtaining the matrices, we start constructing similarity matrices from the adjacency matrices of the properties.

We use the Jacquard similarity function to calculate the similarity matrix for each adjacency matrix. The Jacquard function is expressed in the following equation:

4 $$ J\left( {A,B} \right) = \frac{{M_{11} }}{{M_{01} + M_{10} + M_{11} }} $$

Considering two-feature vectors A and B, where each one contains n elements with values 0 or 1.

- $M_{11}$ means the number of entities that is 1 in both vectors A and B.
- $M_{01}$ means the number of entities that is 0 in vector A and 1 in B.
- $M_{10}$ means the number of entities that is 1 in A and 0 in B.

The Jaccard function for each pair of drugs receives the binary vector of the features of the drugs. Then calculate their similarity using the above formula. For example, to calculate the similarity of two drugs, $d_{i}$ and $d_{j}$, based on the feature of the target proteins. The row $i$ and the row $j$ of the feature matrix are given as input to the Jaccard function.

#### Constructing a heterogeneous network for integrating the data resources

In this stage, we construct an attributed heterogeneous network from the drug_drug edge list. The drug_drug edge list shows interactions between the drugs in the list and specifies the type of relationship. This network has many different edge types. In this network, the nodes refer to drugs and the attributes of the nodes refer to the drug's features. For example, if we consider the drug_pathway similarity matrix as a node attribute, then each vector in this matrix is considered as a node attribute for the corresponding drug. To generate drug representation, we use the attributed heterogeneous network with one of the similarity matrixes as a node attribute in each step. As a result, we will have four networks. The representation vectors are made using network structure and node feature vectors in the embedding process. The embedding process will generate four embeddings’ matrices for each drug for all interaction types. Each matrix has three dimensions indicating the nodes number, the embedding size and the number of edge types.

#### Extracting drugs embedding vectors

In the concept of the neural network, extracting a low-dimensional vector for input entities based on their initial features is called embedding. There are several ways to generate graph embedding. In the proposed approach, we introduce a GNN-based model for learning Attributed Heterogeneous networks to extract the low-dimensional representation of nodes in the network.

In this approach, we adopt an algorithm based on the recent research^44^ to learn the embedding of the attributed heterogeneous network. The proposed algorithm can derive the latent topological properties of the network structure along with the node's attributes. It generates the embedding of every node $v_{i}$ on each edge type $r$ in two parts: base embedding and edge embedding. The model uses the node's attributes and network structure in the transformation function to generate base embedding and edge embedding.

**Figure 9:**
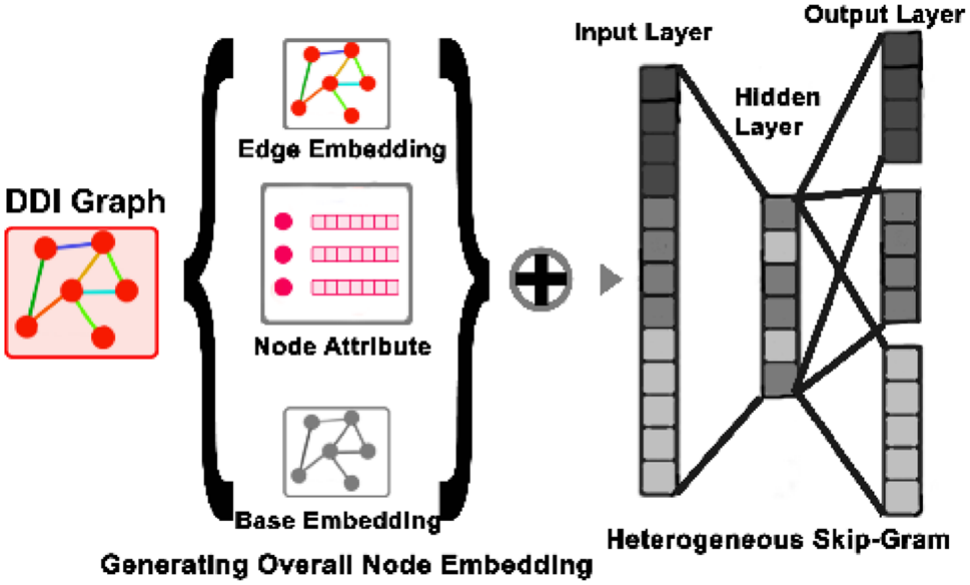
An overview of the embedding model.

The model takes the heterogeneous network and the node's attributes as input. Then the process starts by generating training samples in each edge type using the Random Walk diffusion method. The model creates node sequences using Random Walk and then performs Skip-gram over node sequences to learn embeddings. The model updates to achieve the overall embedding for each node in each edge type. Figure 9 shows an overview of this stage.

Suppose that we have $n$ drugs and $r$ edge type; the drugs embedding using Enzyme similarity matrix as node attributes is $\{ \{ E_{i}^{e} \}^{r} \}^{n}$. The other matrices are $\{ \{ E_{i}^{t} \}^{r} \}^{n} , \{ \{ E_{i}^{p} \}^{r} \}^{n} , \{ \{ E_{i}^{s} \}^{r} \}^{n}$ using Target, Pathway and Chemical structure similarity matrices as node attributes respectively. Generally, the embedding process generates four embedding matrices made from the drug-drug interactions network and four similarity matrices of drug features. Each matrix has three dimensions indicating the nodes number, the embedding size and the number of edge types.

### The second step of the proposed method

**Figure 10:**
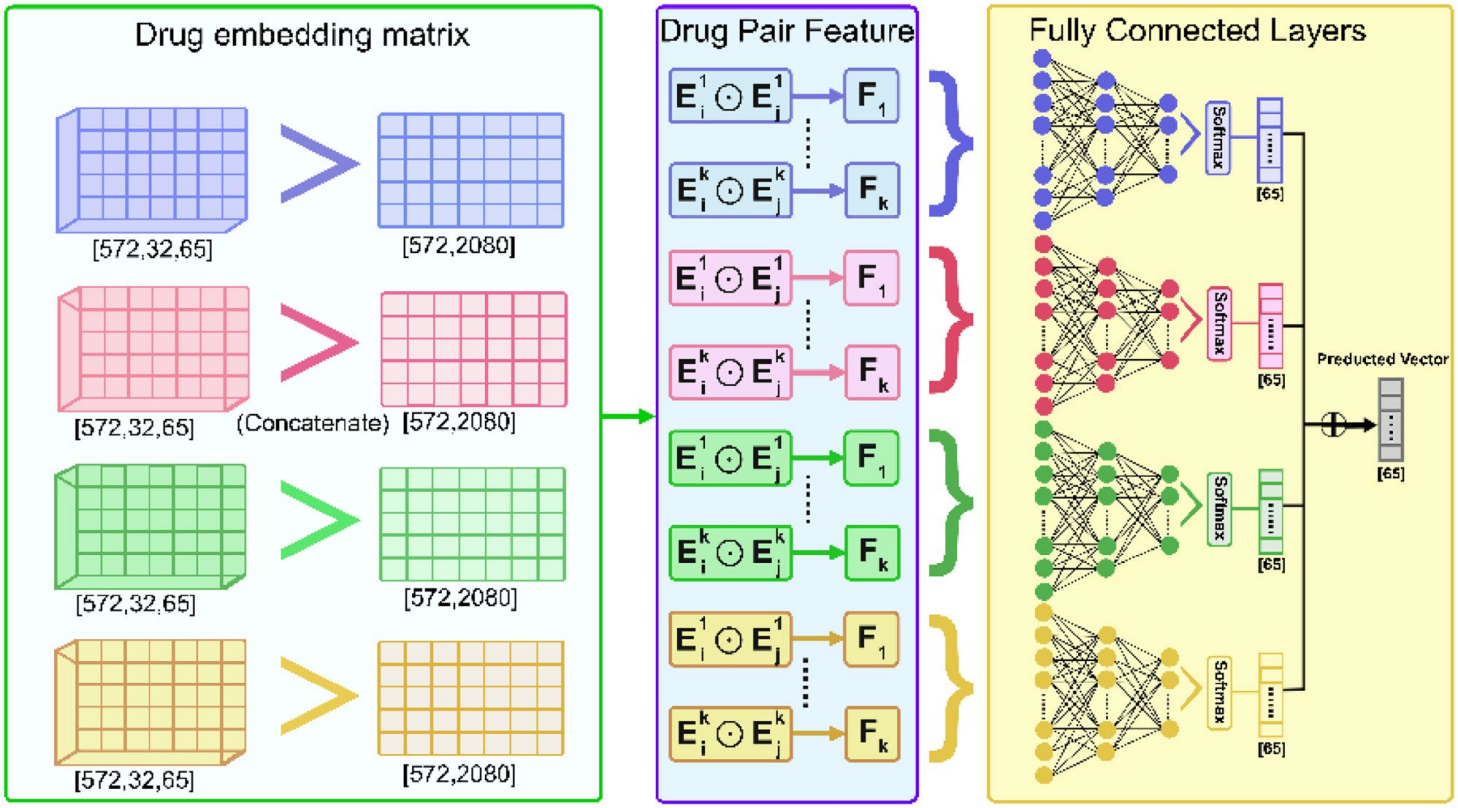
A view of the second stage of the proposed method.

After creating the embedding matrices for drugs, we use a concatenation (aggregation) method to reduce the embedding matrices' dimensions into a one-dimensional feature vector. In a multi fully connected deep learning model, this feature vector is used as an input to predict the DDI types. Figure 10 shows an overview of this process.

#### Dimensions reduction of the embedding matrix

After generating the network embedding matrix, each drug is represented by a two-dimensional matrix. This matrix contains the node (Drug) embedding vectors in each edge type (Interaction type). We use a concatenation method for each drug matrix to merge the drug embedding vectors together. The generated one-dimensional vector represents the embedding vector of the drug $i$ in all edge types. Then, we obtain a feature vector for each drug pair in the DDI list by multiplying the feature vectors of drug $i$ and drug $j$ of the drug pair.

If the embedding matrix of drug $i$ is $M_{i}$ and the vector $v$ in certain edge type $t$ is $v_{i,t}$ then the one-dimensional feature vector $F_{i}$ for drug $i$ is $F_{i} = \left[ {v_{i,1} , \ldots , v_{i,t} } \right]$, and the feature vectors of the drugs pair $k$ is $F_{k} = F_{i} \odot F_{j}$, where $\odot$ is the element-wise product.

#### DDI prediction by a fully connected deep learning network

After producing the four matrices of feature(embedding) vectors in the first step, the fully connected deep learning network is used to perform the prediction task. As shown in Fig. 10, the designed model for the second step consists of four sub-networks. Motivated by the bottleneck-like neural network idea^45^, each sub-network uses one of four matrices of the drug's feature vector as input. The result of these sub-networks is aggregated to achieve the final result. We use several hidden layers in the networks and batch normalization layers^46^ between them. Then a softmax layer is employed for prediction in these sub-networks. Finally, to enhance generalization ability and avoid over-fitting, we add dropout layers to the networks^47^. We adopt (ReLU)^48^ as an activation function in the networks. Here, the outputs of the sub-networks are merged by calculating the average and producing the final prediction.

We choose the cross-entropy loss function and utilize the Adam optimizer with the default parameters for the optimization algorithm. To control over-fitting while speeding up the training process, we use the early-stopping approach^49^. With this approach, if no improvement is observed in 10 epochs, the training automatically stops.

## Data Availability

The datasets and codes using in this study are available in https://github.com/Mohammad-Hussain95/GNN_DDI.
